# The 4-Level Approach: Prevention of Suicidal Behaviour Through Community-Based Intervention

**DOI:** 10.3389/fpsyt.2021.760491

**Published:** 2021-12-02

**Authors:** Ulrich Hegerl, Ines Heinz, Ainslie O'Connor, Hanna Reich

**Affiliations:** ^1^Johann Christian Senckenberg Distinguished Professorship, Department for Psychiatry, Psychosomatics and Psychotherapy, Goethe University, Frankfurt, Germany; ^2^German Depression Foundation, Leipzig, Germany; ^3^German Alliance Against Depression, Leipzig, Germany; ^4^European Alliance Against Depression e.V., Frankfurt, Germany; ^5^Depression Research Centre of the German Depression Foundation, Department for Psychiatry, Psychosomatics and Psychotherapy, Goethe University, Frankfurt, Germany

**Keywords:** suicide prevention, depression, 4-level approach, multilevel intervention, community-based intervention

## Abstract

Due to the many different factors contributing to diagnostic and therapeutic deficits concerning depression and the risk of suicidal behaviour, community-based interventions combining different measures are considered the most efficient way to address these important areas of public health. The network of the European Alliance Against Depression has implemented in more than 120 regions within and outside of Europe community-based 4-level-interventions that combine activities at four levels: (i) primary care, (ii) general public, (iii) community facilitators and gatekeepers (e.g., police, journalists, caregivers, pharmacists, and teachers), and (iv) patients, individuals at high risk and their relatives. This review will discuss lessons learned from these broad implementation activities. These include targeting depression and suicidal behaviour within one approach; being simultaneously active on the four different levels; promoting bottom-up initiatives; and avoiding any cooperation with the pharmaceutical industry for reasons of credibility.

## Introduction

**Figure 1:**
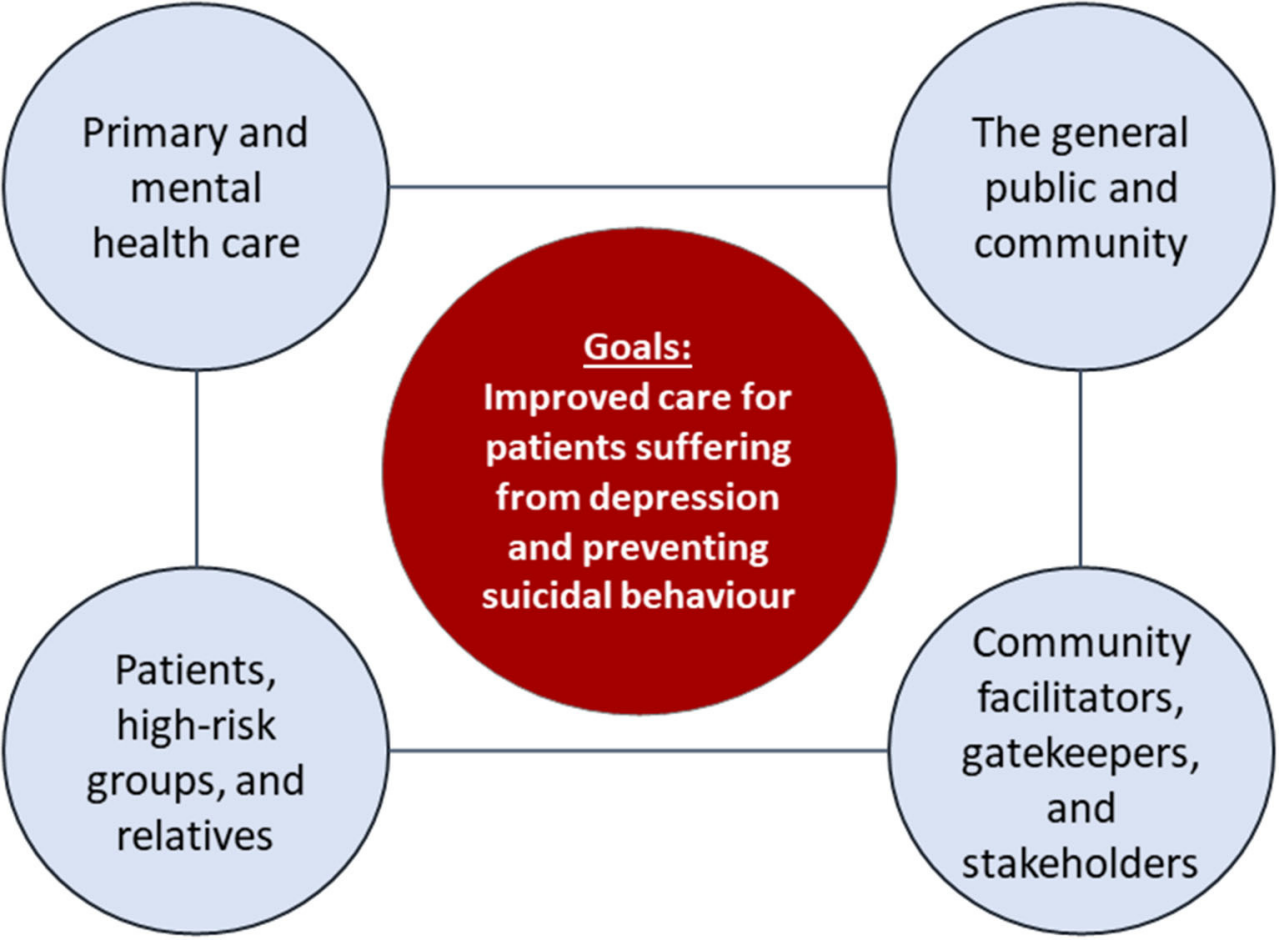
The 4-level intervention concept (reproduced with courtesy of the European Alliance Against Depression e.V.). *Level 1—Interventions for primary and mental health care professionals:* training sessions and practise support for General Practitioners (GPs) and mental health care professionals regarding the diagnosis and treatment of depression and managing suicidality, including iFightDepression®, an e-learning guided web-based intervention tool for their patients. For more details on the iFightDepression®-tool please refer to Oehler et al. (8). *Level 2—Interventions for the general public and community:* a professional public relations campaign, addressing the general public with the key messages “Depression can affect anyone,” “Depression is a real disease,” and “Depression can be treated.” Generally, regional suicide preventive activities are started by an opening ceremony in a public place, together with a famous spokesperson. *Level 3—Interventions for community facilitators, gatekeepers, and stakeholders:* training sessions concerning the topics depression and suicide risk for community facilitators and gatekeepers such as geriatric care givers, priests, pharmacists, police, and journalists. These trainings involve role-plays on topics such as how to explore suicide risk and mental ill-health. Cooperation with journalists is based on a media guide with the aim to reduce copycat suicides (Werther effect). *Level 4—Interventions for patients, high-risk groups, and relatives:* support for patients and their relatives by providing information on depression and the iFightDepression® tool, supporting other self-help activities and providing an emergency card for people after an attempted suicide. An important aspect of suicide prevention, reducing access to lethal means, is considered in different levels (e.g., smaller package size of lethal drugs, identification and securing “hot spots” for suicide in the community). For more details please see Hegerl et al. (9).

Given the variety of contributing factors and the complexity of suicidal behaviour, multifaceted community-based interventions combining different suicide preventive measures [e.g., (1–3)] are amongst the most promising approaches to prevent suicidal behaviour (attempted and completed suicides) (4, 5), as no single intervention for suicide prevention is at a clear advantage (6). The EAAD (European Alliance Against Depression e.V.) 4-level-intervention concept (7) combines in a coherent manner several interventions, for which at least some evidence of suicide preventive effects exists (6). These encompass education of primary care and mental health professionals, gatekeeper trainings, digital interventions for depression, guidelines for media, a public awareness campaign, and a reduction of access to lethal means. In addition, the 4-level approach combines the two, partly overlapping aims of improving care for people with depression and preventing suicidal behaviour. The advantages of these dual aims will be discussed below. Intervention activities are implemented simultaneously at four levels in the community (see Figure 1).

Evidence for the efficacy of this 4-level intervention concept regarding the prevention of suicidal behaviour (attempted and completed suicides as primary outcome) is summarised in Table 1. Based on a priori defined primary outcome, and a baseline and a control region, clear support for preventive effects on suicidal acts and the sustainability of these effects was provided by the model project, Nuremberg Alliance against Depression (NAD) [(1, 14), see Table 1]. The large international controlled intervention study, OSPI-Europe [Optimising Suicide Prevention Programmes and their Implementation in Europe, (15)], replicated the preventive effects of the 4-level intervention concept in one region in Portugal, but did not find statistically significant effects in regions in Germany, Ireland, and Hungary (9). Reasons for these mixed results are discussed by Hegerl et al. (9). When considering completed suicides, evidence for the anti-suicidal efficacy of the 4-level intervention concept has been provided by studies from Germany and Hungary (see Table 1). Reliable data for effect sizes of single intervention elements that are applied within the 4-level intervention concept are difficult to obtain, as synergistic effects are generated by simultaneous activities on all four levels (12, 16). A variety of partly unforeseeable factors [e.g., national election interfering with the PR-campaign, flooding catastrophe in an intervention region interfering with the implementation, differences in intervention intensity; for discussion see (13)] have introduced a considerable variance to observed effect sizes for the 4-level intervention as a whole (9).

**Table 1:** Evidence supporting the 4-level intervention concept (controlled studies only).

**Study**	**Intervention region and period**	**Primary outcomes**	**Results**
Hegerl et al. (1)	Nuremberg, Germany (ca. 500,000 inhabitants) 2001–2002	Number of suicidal acts (attempted + completed suicides) compared to both a baseline year (2000) and a control region (Wuerzburg, 270,000 inhabitants).	A significant reduction in the number of suicidal acts (−21.7%) was found in Nuremberg compared to both a baseline year (2000) and the control region. Compared to rates in the baseline year 2000, reductions of suicidal acts was sustainable in the follow-up year (2003, −32.4%).
Hübner-Liebermann et al. (10)	Regensburg, Germany (ca. 150,0000 inhabitants) 2003–2007	Suicides rates during the 5 years of 4-level interventions were compared to those in the preceding 5 years, as well as to changes in two control regions and the nation-wide overall suicide rate.	A significant decrease of suicide rates was observed compared to the baseline and two control regions, and to the national trend.
Székely et al. (11)	Szolnok, Hungary (ca. 70,000 inhabitants) 2004–2006	Suicide rates during the 2 years of 4-level intervention and one follow-up year were compared to the rates in a control region (Szeged, ca. 160,000 inhabitants) and to national suicide rates.	The decrease of annual suicide rates in Szolnok after the onset of the intervention was significantly stronger than the one observed in the control region (*p* = 0.0015) and the whole country (*p* = 0.017).
OSPI-Europe (“*Optimising Suicide Prevention Programmes and their Implementation in Europe”)* Main outcome paper: Hegerl et al. (9) Process analysis: Harris et al. (12, 13)	1. Amadora (Portugal, ca. 170,000 inhabitants) 2. Leipzig (Germany, ca. 500,000 inhabitants) 3. Limerick (Ireland, ca. 190,000 inhabitants) 4. Miskolc (Hungary, ca. 170,000 inhabitants) 2008–2011	Number of suicidal acts (attempted + completed suicides) compared to a 1-year baseline in the intervention regions and the respective control regions (Almada, Portugal, ca. 165,000 inhabitants; Magdeburg, Germany, ca. 230,000 inhabitants; Galway, Ireland, ca. 240,000 inhabitants; Szeged, Hungary, ca. 170,000 inhabitants).	Significant intervention effects compared to baseline and corresponding changes in the control region were observed in Portugal. No such effects were found in Germany, Ireland, and Hungary. Major intervening factors identified by systematic process analysis performed within the OSPI project are discussed (e.g., flooding and elections in Hungary, celebrity suicide of Robert Enke in Germany, negative societal impact of the economic recession in Ireland). Better recognition of suicidal acts due to the awareness campaign might have introduced a bias in the official numbers of suicidal acts in the intervention regions.

## Lessons Learned

Since 2008, the 4-level intervention concept has been implemented in more than 120 regions in Germany and a further 15 countries in Europe, plus Australia, and North and South America. Extensive practical experience in the implementation of these community-based interventions in different countries and thus health care systems, has been accumulated. In addition, systematic process evaluation and implementation research was conducted within the OSPI-Europe project addressing efficiency, capacity building processes and synergistic effects that unfold through the interplay between the four single intervention levels (12, 13). In the following, six lessons learned from both the practical experience and the process analyses will be summarised:

### Combine the Targets of Improving Care for Depression and Suicide Prevention

The 4-level intervention concept combines the following two, partly overlapping aims: (i) to improve care for people with depression and (ii) to prevent suicidal acts. At a first glance, this approach may appear unfamiliar, as it is generally different research groups working on suicide prevention to those dealing with depression. However, combining these aims makes sense for several reasons:

➢ Depression as a very prevalent and severe disorder is the mental disorder associated with by far, the largest burden of disease in the population (17). Depression is also a key causal factor concerning suicidal behaviour (18). Of course, other mental disorders such as schizophrenia, drug and alcohol addictions, bipolar affective disorders, eating disorders or personality disorders are also associated with an increased suicide risk. However, to address all these disorders with sufficient intensity might be very challenging, as their manifestation, prevention and treatment options or treatment gaps differ considerably and therefore require specific interventions.
➢ Depression affects directly or indirectly more than 50% of the population and a public relations campaign on depression is likely to have a greater resonance in the community than a pure suicide prevention campaign.
➢ Campaigns focusing solely on suicide may on the one side be beneficial by destigmatizing suicide and improving help-seeking of those at risk, but on the other side may even increase suicide rates *via* a variety of possible mechanisms. For instance, destigmatization and normalisation of suicidal behaviour may reduce the threshold concerning suicide. Also, uncontrollable secondary reporting about the topic suicide, in social media for example (e.g., discussion of lethal methods), may have unwanted effects by increasing the cognitive availability of certain suicidal methods (19), by elevating suicide capability and knowledge of lethal means due to increased internet searches (20) or by inducing the Werther effect [copycat suicides (21–23)]. Whilst talking about suicide responsibly is not likely to increase the risk of suicide and active exploration by health professionals is strongly recommended; the question concerning the benefit-risk ratio of large anti-suicide public relation campaigns addressing the general public is difficult to answer, as outlined above. Great importance has to be placed on how such a campaign is designed (communication style, approach, and platforms). By combining the aims of improving care for depression and suicide prevention, the focus of campaigns can be shifted to depression when addressing the general public, and more to suicide prevention when addressing general practitioners, mental health professionals, and community facilitators.
➢ Depression and suicide prevention are substantial, yet overlapping public health concerns, both with a huge room for improvement. Combining and addressing them within one intervention concept is highly cost effective. A study modelling prospectively the value of the implementation of the 4-level intervention concept demonstrated its cost-effectiveness at the population level and resulted in cost-savings due to averted suicide deaths and a reduction in life years lost (24).

### Be Simultaneously Active at All Four Intervention Levels

Combining different interventions has not only added, but also created synergistic effects (12). For instance, the public health campaign within the EAAD 4-level intervention starts with a public launch ceremony in the respective community. This facilitates the uptake and visibility of trainings available for general practitioners, community facilitators and journalists, and leads to increased media reporting. For those affected by depression or other mental disorders, the public campaign may increase knowledge about the symptoms of depression, treatment options and reduce the perceived depression stigma, thus improving help seeking behaviour and lessening the isolation which contributes to despair and suicide risk (25). It also helps general practitioners to confront a patient presenting mainly somatic symptoms with a possible mental disorder. In addition, catalytic effects can be triggered: the community-based intervention within the 4-level approach has been observed to stimulate additional activities that add value to, but are nevertheless external to, planned activities. An example is improvement of intersectoral cooperation between mental health professionals in the community [see (12)]. It is important to become simultaneously active with sufficient intensity on all four intervention levels to create a critical mass of activities as well as synergistic and catalytic effects.

### Keep the Balance Right Between a Bottom-Up and Top-Down Approach

A strong bottom-up approach, inherent to the EAAD 4-level-intervention concept, proved to be helpful for successful implementation and to create an ownership feeling at the community level (1). Therefore, it is important to go out into the community and organise meetings for example, not in the leading academic institution, but preferably in a known, shared public space. This bottom-up approach also improves sustainability, whereas interventions financed top-down risk being perceived as a time-limited project associated with a lower level of ownership from the community. However, in countries with less developed social capital and civic engagement, a more pronounced top-down element might be helpful and necessary for successful implementation.

Although the bottom-up approach is encouraged, as it develops a strong sense of community ownership, being cognisant of national policy, legislation and actions related to mental health can be beneficial. Understanding this context can provide opportunities, for example to engage in a national narrative around suicide prevention to the benefit of local awareness or to leverage funding opportunities created by a national mental health strategy (5).

### Work Independently of the Pharmaceutical Industry

Antidepressants and other pharmacotherapies play an important role in the treatment of psychiatric disorders associated with a high suicide risk. Lithium has been shown to have specific anti-suicidal effects in unipolar depression (26). Therefore, promoting guideline-oriented psychopharmacological treatment is an important topic in the training of health professionals. However, for credibility reasons and to avoid conflict of interests, the pharmaceutical industry should not be involved in the funding and execution of the 4-level intervention activities.

### Start Locally: Develop a Model Project and Then Transfer to Further Regions in That Country

Experience from Germany and other countries shows that when the intervention materials have been developed and the 4-level intervention concept has been implemented in a model project [in Germany it was the Nuremberg Alliance against Depression, (1, 14)], other regions might be interested to also implement this intervention. As the local alliances are the ones that carry out activities on each of the 4 levels, it makes sense to create a learning network of all implementing regions/partners with the aim to exchange experiences, support each other with advice and to further improve or complement the intervention material catalogue. Regions intending to implement an alliance and those with newly established alliances benefit from the experience of the partners in the EAAD network and the support of a national coordinator. More than 85 regions have started their own regional 4-level interventions in Germany (27) and in order to promote the exchange of experiences, the partners from the different regions are invited by the national coordinator three times per year to a meeting. Typical topics discussed at these meetings are how best to design the organisational and operational structure of the alliance and how to integrate its activities within the local community and service environment, how to professionalise fundraising (e.g., How do I identify and approach a potential sponsor? Which institutions typically support a local alliance in other regions?), how to organise a press conference, or how to optimise the impact of PR-campaigns with limited resources.

Depending on the resources available, the evaluation of a project applying the 4-level intervention concept comprises the documentation of intervention activities, the primary outcomes (suicidal behaviour), and a variety of intermediate outcomes, such as evaluations of trainings of general practitioners and community facilitators (28–30), changes in stigma and knowledge in the general population (25, 31), and changes in media coverage (32).

### Keep Your Borders

The intervention region should be clearly defined by postal codes and the start of the intervention should always be marked by a prominent opening ceremony. Sometimes, there can be a tendency or desire to extend the campaign from depression to mental health in general. As stated above, it has been crucial to focus on depression rather than all mental illnesses as it allows the interventions to be sufficiently specific, ensuring they are seen, remembered, and effective. We therefore advise against expanding the intervention concept to include other mental disorders (e.g., anxiety disorders) or non-clinical aspects such as well-being and lifestyle (e.g., stress and burnout). The same applies to debates about the geographical size of intervention regions. Envisioning alliances on a bigger geographical level (e.g., federal states) might be tempting. However, a more regional approach is helpful for creating a feeling of ownership and for identifying local stakeholders that become part of “their alliance against depression.” In order to be clearly perceived and identifiable, it is genuinely important that the community-based 4-level intervention keeps its borders in terms of geographical reach, time and content.

## Outlook

Combining the targets *Depression* and *Suicide* in the 4-level intervention concept has proven to have major advantages and allows a shift in focus between the two aspects depending on the target group.

The 4-level intervention concept developed by the EAAD is the most broadly implemented and evaluated community-based program targeting both depression and suicide worldwide. It has been recognised by numerous awards and in 2019, was voted by European Union Member States as best practise in mental health at the European Commission's Joint Research Centre. A comprehensive catalogue of intervention and evaluation materials has been established and optimised over the last 20 years. Among the intervention materials is the iFightDepression® tool, a guided, web-based intervention for mild to moderate depression and dysthymia (8). This self-management tool is based on the principles of Cognitive Behavioural Therapy (CBT) and is available in 12 languages, including an Arabic version. Core 4-level intervention materials have been translated to more than ten different languages for implementation in EAAD member countries, a number that will be expanded during the European Union funded project “EAAD-Best” (European Union Health Programmes Fund, 2021-2023). This new project is focused on implementing the 4-level intervention concept in five Eastern and Southern European countries (Bulgaria, Estonia, Greece, Italy, and Poland) that are yet to have a national suicide strategy, and on expanding interventions to new regions in three EAAD-member countries (Hungary, Ireland, and Spain). Experience to date shows that all intervention materials can be easily adapted to different cultures and health care systems. The European Alliance Against Depression (www.eaad.net) provides support for all regions in and outside of Europe that intend to start their own 4-level intervention programmes.

## Author Contributions

All authors listed have made a substantial, direct, and intellectual contribution to the work and approved it for publication.

## Conflict of Interest

UH was a member of Janssen's Advisory Board. The remaining authors declare that the research was conducted in the absence of any commercial or financial relationships that could be construed as a potential conflict of interest.

## Publisher's Note

All claims expressed in this article are solely those of the authors and do not necessarily represent those of their affiliated organizations, or those of the publisher, the editors and the reviewers. Any product that may be evaluated in this article, or claim that may be made by its manufacturer, is not guaranteed or endorsed by the publisher.
